# A44 ENDOSCOPISTS PERFORMING ADVANCED UPPER GI ENDOSCOPY ARE AT HIGHER RISK OF FACIAL BACTERIAL CONTAMINATION

**DOI:** 10.1093/jcag/gwae059.044

**Published:** 2025-02-10

**Authors:** M Baeg, S Ko

**Affiliations:** Gastroenterology, Jeju National University Hospital, Jeju, Jeju-do, Korea (the Republic of); University of Toronto Temerty Faculty of Medicine, Toronto, ON, Canada

## Abstract

**Background:**

Gastrointestinal (GI) endoscopy has been recognized as putting endoscopists at risk for bacterial exposure. However, whether longer, more advanced procedures such as endoscopic submucosal dissection (ESD) or endoscopic retrograde cholagiopancreatography (ERCP) confer greater risk has not been investigated.

**Aims:**

This study aimed to investigate the degree of exposure on endoscopists who performed ESD or ERCP and find out if longer time or procedure type are risk factors for exposure.

**Methods:**

This was a prospective study examining the facemasks of endoscopists who performed ESD or ERCP at a university hospital, with 40 procedures planned for each procedure. The face shield was swabbed in a standardized way before and at the end of the procedure. Swabs were also taken from face shields placed 2 meters away on the endoscopy suite wall, patient recovery room wall, and after direct contamination by the endoscope immediately after the procedure. The swabs were cultured for 48 hours, and growth was reported as no growth or by number of colony-forming units (CFUs). The groups were compared for +CFU rate. Risk of contamination was analyzed by procedure time and type.

**Results:**

55 ESDs and ERCPS were performed during the study dates. It was decided to terminate the study early, due to the COVID-19 situation, as facemasks were deemed too important to be used for walls. Excluding one facemask group in which the endoscopist’s facemask was exposed before the procedure, 54 groups of masks were analyzed. There were 33 ESDs and 21 ERCPS performed, with 26 upper GI and 7 lower GI ESDs. A cut-off of 30 CFUs were determined as indication of definite exposure. Interestingly, none of the 7 lower GI ESDs resulted in any exposure, so it was decided to analyze only the 47 orally inserted procedures. There were 9 positive exposures, 6 ESDs (23.1%) and 3 ERCPs (14.3%). Mean procedure time was 30.3 ± 15.2 minutes. Procedure time (Odds ratio 1.014, 0.968-1.061, P=0.564) and type (ERCP compared to ESD, OR 0.556, 0.121-2.553, P=0.556) were not positive risk factors for contamination. The most commonly cultured pathogens were Staphylococcus, Streptococcus, Bacillus, and Moraxella species.

**Conclusions:**

This study confirmed that advanced procedures such as upper GI ESDs and ERCPs have a high risk of facial exposure. Facemasks and other protective equipment should be used during GI endoscopy.

Basic Characteristics of Endoscopy

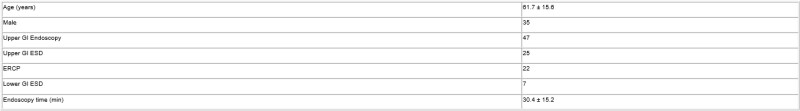

ESD - endoscopic submucosal dissection, ERCP - endoscopic retrograde cholangiopancreatography

**Funding Agencies:**

None

